# Plasma homocysteine level and trajectories in association with longitudinal increase in plasma neurofilament light among urban adults

**DOI:** 10.1007/s11357-025-01567-z

**Published:** 2025-02-19

**Authors:** May A. Beydoun, Nicole Noren Hooten, Michael F. Georgescu, Hind A. Beydoun, Marie T. Fanelli-Kuczmarski, Jordan Weiss, Michele K. Evans, Alan B. Zonderman

**Affiliations:** 1 Laboratory of Epidemiology and Population Sciences, NIA Biomedical Research Center/NIH/IRP, Baltimore, MD 21224 USA; 2 VA National Center On Homelessness Among Veterans, U.S. Department of Veterans Affairs, Washington, DC USA; 3 Department of Management, Policy, and Community Health, School of Public Health, University of Texas Health Science Center at Houston, Houston, TX USA; 4 Stanford Center on Longevity, Stanford University, Palo Alto, California, CA USA

**Keywords:** Plasma Neurofilament light, Homocysteine, One-carbon metabolism, Urban adults, Race, Cognition

## Abstract

We tested whether homocysteine (Hcy) was linked to longitudinal change in plasma neurofilament light chain (NfL) overall and differentially across sex and race in a sample of middle-aged urban adults. We used data from the Healthy Aging in Neighborhoods of Diversity across the Life Span (HANDLS) study [*n* = 690, Age at visit 1 (v_1_:2004–2009): 30-66y, 42.1% male, 55.4% African American] to evaluate relationships between NfL levels and both visit 1 Hcy (Hcy_v1_) and time-dependent Hcy concentrations (Hcy_td_). Specifically, we examined the relationship between these measures and visit 1 NfL (NfL_v1_) as well as annualized change in NfL (δNfL) over the study period of ~ 8y [2004–2009 (v_1_), 2009–2013 (v_2_) and/or 2013–2017 (v_3_)] using mixed effects linear regression models. Hcy_v1_ was positively associated with NfL_v1_, but not with δNfL. However, when Hcy_td_ was considered along with time-dependent covariates on multiple-imputed data, both NfL_v1_ and δNfL were associated with Hcy_td_, with some significant sex difference in the longitudinal association, whereby this relationship was stronger among males. The Hcy exposure obtained from group-based trajectory models, indicated that individuals belonging to the "High increasing" group were consistently associated with both higher NfL_v1_ and faster increase in NfL (overall, fully adjusted, High *vs*. Low: γ_0gbtm_ =  + 0.226 ± 0.058, P < 0.001; γ_1gbtm_ =  + 0.022 ± 0.007, P < 0.010). Dynamic Hcy exposures (Hcy_td_ and Hcy_gbtm_) were associated with faster rate of increase in NfL over time, reflecting potentially faster rate of axonal degeneration. Further studies are needed in comparable populations to replicate our findings.

**Supplementary Information:**

The online version contains supplementary material available at 10.1007/s11357-025-01567-z.

## Introduction

Homocysteine (Hcy), a sulfur-containing amino acid, is influenced by various factors including dietary intake, genetic factors, lifestyle, age, sex, disease states, and dietary supplements[1]. Elevated Hcy can lead to adverse health effects, such as increased risk of atherosclerosis, thrombosis, and neurodegenerative diseases[2]. Factors affecting Hcy levels include deficiencies in folate, vitamin B6, and B12, genetic polymorphisms in Methylenetetrahydrofolate reductase (MTHFR), smoking, alcohol consumption, and physical activity[3]. Elevated Hcy can also lead to oxidative stress, endothelial dysfunction, and inflammation[4]. Hyperhomocysteinemia, characterized by elevated Hcy in the bloodstream, is recognized as a separate risk factor for peripheral vascular, brain, and cardiovascular illnesses [5]. Consequently, there has been conjecture on the potential influence of hyperhomocysteinemia on the cognitive abilities of elderly individuals. Multiple studies have also established an association between elevated levels of Hcy and a heightened susceptibility to developing Alzheimer’s Disease (AD) or dementia from any cause [6–12]. Research has shown that Hcy has a specific impact on cognitive performance [13]. Due to potential regional variations in the brain, Hcy has been linked to increased occurrences of white matter hyperintensities and brain atrophy[14–16]. In addition to Hcy’s link to cardiovascular disease, in vitro studies have shown that Hcy possesses neurotoxic and excitotoxic properties, which provide biological plausibility for its direct causal effect on cognition. Understanding the interaction between Hcy and NfL biomarkers can help develop improved diagnostic and treatment approaches for neurological diseases.

Similarly, neurofilament light chain (NfL) is a neuronal cytoskeleton protein, which is a sensitive indicator for neuronal damage in neurological disorders ranging from multiple sclerosis to AD [17, 18]. Cytoskeletal proteins are often discharged into extracellular space in response to axonal injury and neurodegenerative disorders, cerebrospinal fluid (CSF), ultimately making their way to the blood stream at lower concentrations[19, 20]. In fact, plasma NfL levels were also linked to cognitive decrements in non-demented persons [21, 22] and its measurement in blood is thought to be less intrusive than its direct assessment in the CSF[23, 24]. CSF and plasma NfL have both been associated with lower cognitive performance, although findings for plasma NfL remain mixed. [25].

Consequently, plasma concentrations of homocysteine and neurofilament light chain (NfL) are biomarkers that are crucial in understanding neurological health and illness. Homocysteine, a sulfur-containing amino acid, is linked to cardiovascular diseases, cognitive decline, and neurodegenerative disorders[5]. Nevertheless, the question as to whether age-related elevated levels of blood Hcy over time is an independent risk factor for the of NfL growth over time remains unknown, with limited research suggesting a positive association [26–29]. These associations may differ by sex and race particularly considering sex- and race-specific relationships of plasma NfL with adverse cognitive performance.

Furthermore, prior research has demonstrated a clear direct association between plasma NfL and unfavorable neurocognitive outcomes. This direct relationship has also been detected for hyperhomocysteinemia. Consequently, it is hypothesized that plasma NfL and blood Hcy concentrations may also have a direct relationship, which would imply that, when evaluating the therapeutic usefulness of plasma NfL as a precursor to neurodegeneration, elevated Hcy in blood must be taken into consideration as a possible confounding factor. More importantly, this relationship may also suggest that the total effect of blood Hcy on cognitive performance over time, may be mediated through plasma NfL.

The present study investigated the longitudinal associations of initial-visit plasma Hcy concentration with initial visit NfL (NfL_v1_) and longitudinal change in NfL (δNfL) over time, even after adjustment for key confounding factors and stratification by sex and race. The study also assessed whether time-dependent values and trajectories of plasma Hcy concentrations over time were associated with these two outcomes of interest.

## Materials and methods

### Study design

The current longitudinal observational study, Healthy Aging in Neighborhoods of Diversity Across the Life Span (HANDLS), examines health inequalities in aging and age-related conditions among White and African American community-dwelling middle-aged adult women and men living above or below poverty [30–38]. The study was initially done in Baltimore, MD, and consisted of two phases: Phase I included home visits for recruiting, consent, screening, and a 24-h dietary recall. Furthermore, at the second phase, in-person full physical health evaluation on Medical Research Vehicles (MRV) coupled with a second 24-h dietary recall were carried out. Participants were asked to additional in-person visits to collect fasting blood samples and undergo physical assessments, following a similar study protocol. The three study visits included in our present analysis were completed during time frames of 2004–2009 (visit 1, v_1_), 2009–2013 (visit 2, v_2_) and 2013–2017 (visit 3, v_3_). The National Institute of Environmental Health Sciences' Institutional Review Board approved the study procedure, and all participants signed a written informed consent form.

## Study sample

**Fig. 1:**
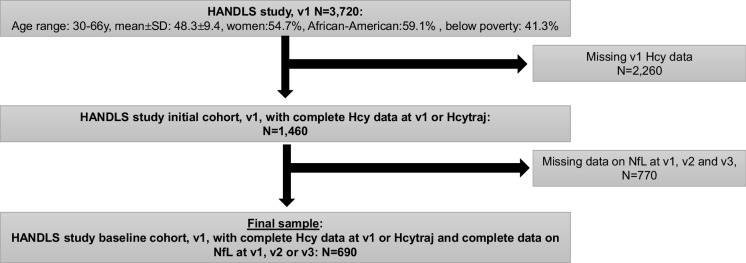
Participant Flowchart. Abbreviations: HANDLS = Healthy Aging in Neighborhoods of Diversity Across the Life Span; NfL = plasma Neurofilament Light; v_1_ = Visit 1; v_2_ = Visit 2; v_3_ = Visit 3

Plasma NfL concentrations were measured repeatedly, up to three times between visits 1 and 3. In our main analyses, we selected the sample based on the availability of any of these 3 repeats as well as v_1_ Hcy exposure. Of the 3,720 HANDLS individuals who were initially recruited, n = 1,460 had information about visit 1 Hcy and complete data used for Hcy_traj_. Of this group, n = 690 had additionally complete v_1_, v_2_, and/or v_3_ data on plasma NfL (Fig. 1). The percentage of participants with household incomes below poverty was lower in the final selected sample in comparison to the initial sample (p < 0.001, χ^2^ test) that had incomplete data for our study (28.7% vs. 44.1%). This pattern was similar for the percentage African American (55.4% vs. 59.1%, p = 0.028, χ^2^ test). **Supplementary method 1** details sample selection for the NfL data.

## Plasma neurofilament light

Fasting blood samples were collected in the morning from participants into EDTA d collection tubes. Blood was processed as previously described[21, 39] and plasma was aliquoted and stored at −80 °C till use. The levels of NfL in plasma were measured using the Simoa® NF-light Advantage Kit developed by Quanterix (Billerica, MA, USA), as previously described in detail[21, 39].

## Plasma homocysteine

For the purpose of collecting serum, blood samples were obtained in the morning using BD Vacutainer® serum separator tubes after fasting. The samples were subjected to centrifugation at room temperature with a force of 1142 g for a duration of 15 min, with the brake engaged. Serum was then aliquoted and stored at −80 °C until use. Hcy was quantified by Aeon Technologies, LLC. All serum samples passed quality assessments using a Alinity i analyzer. The Alinity i Homocysteine test was utilized to measure the concentration of Hcy, with a range of detection from 1.00 to 50.00 mmol/L. The primary independent variable in this study was the measurement of Hcy at v_1_, which was transformed using the natural logarithm (LnHcy_v1_). Group-based trajectory models or GBTM were applied to time-dependent LnHcy over 3 waves of data, which is a set of statistical methods used to identify subgroups within a population that share similar developmental or temporal patterns over time. A STATA plugin was utilized to ascertain these group trajectories over time (https://www.andrew.cmu.edu/user/bjones/) [40]. This approach utilizes the maximum likelihood method and a multinomial modeling strategy to accurately estimate the parameters of the model. The quasi-Newton method was employed to maximize the outcomes [40, 41]. The analyses yielded group-based trajectories across time, along with 95% confidence intervals (CI), defined by an intercept and a slope for a linear trajectory group and other parameters added to linear age for higher-order trajectory groups (e.g. quadratic). The chosen outcomes were modeled using a censored normal distribution. Up to 3 groups extracted from the GBTM were specified with preference given to linear models for ease of interpretation. Comparison with higher order models (quadratic, cubic) per group were made using the Bayesian Information Criterion (BIC).

## Covariates

The study considered various variables as potential confounders, including sex and race, age, poverty status[42], and educational attainment. Sex (Male vs. Female) and race (African American vs. White) were the primary effect modifiers, while lifestyle behaviors like smoking status, illicit drug use, the Healthy Eating Index 2010 for diet quality[43], total energy intake, and the Center for Epidemiological Studies-Depression (CES-D) total score for depressive symptoms[44] were considered as potential confounders. Poverty status was determined through criteria from the Department of Health and Human Services poverty levels based on household income and total household size [45].These factors were intentionally collected to understand the potential influence of these factors on Hcy and plasma NfL.

Health-related covariates based on medical examination and history, blood measurements and medication use were also considered among potential confounders. Measures included self-reported hypertension (no, yes), self-reported diabetes (no, pre-diabetes, diabetes), hypercholesterolemia (using measured total cholesterol and statin use), and self-reported cardiovascular disease (any of angina pectoris, atrial fibrillation, myocardial infarction, coronary artery disease or congestive heart failure). Trained examiners utilized conventional methods to evaluate several health-related factors such as body mass index (BMI) (measured weight (in kilograms) divided by the square of the measured height (in meters), waist-to-hip ratio, radial pulse rate (beats per minute), and systolic and diastolic blood pressure (measured in millimeters of mercury). Specifically, blood pressure was assessed using a mercury sphygmomanometer, and the average of the systolic and diastolic values from both the left and right sides were utilized in this research. The overall allostatic load (AL) score based on cardiometabolic health risk factors was calculated using a previously documented computational method^33^. Specifically, AL reflects risk indicators related to cardiovascular health, metabolic health, and inflammatory markers and the total score could range between 0 and 9, as summarized in Table S1 [21, 39]. Contract laboratories measured total cholesterol, HDL-cholesterol, hsCRP, albumin, and glycosylated hemoglobin levels. Less than 5% of observations required multiple imputation per study covariate [21, 39].

## Statistical analysis

We analyzed data for our present study using Stata release 18[46]. Differences in key variables were tested across race and sex, mainly by using bivariate linear, logistic, and multinomial logit models. These models were further adjusted for demographic and socioeconomic characteristics, including age, sex, race, and poverty status. The primary hypotheses were tested using mixed-effects linear models, with the dependent variable being the longitudinal measurement of plasma NfL. The main independent variable was the measurement of Hcy at v_1_ (2004–2009). The modeling strategy involved two sets of models. These models adjusted for potentially confounding covariates in an incremental way. Such covariates, which were deemed potential confounders in the relationship between v_1_ Hcy exposure and plasma NfL, were also included as main effects and interacted with time on study (in years), denoted as *TIME*.

Model 0 was the unadjusted model, while Model 1 was the reduced model, including sex, baseline age, poverty status, race and educational attainment. Model 2 further adjusted for lifestyle and health-related covariates. For maintaining an equal sample size across models, we carried out multiple imputations for main covariates that had missing data. This was achieved using chained equations, with 5 imputations and 10 iterations [47, 48]. All covariates were included simultaneously in the estimation procedure, following the approach of prior investigations [47, 48]. We subjected plasma NfL to Log_e_ transformation in a uniform manner across models. Such transformation has often been used in prior investigations for the purpose of normalization (e.g. [49]). The study also focused on differences based on race and sex in the association between Hcy at v_1_ and v_1_ NfL. Separate models were used to evaluate the interaction between Hcy and race, as well as Hcy and sex through inclusion of 2-way interaction terms. Each of these models also included an examination of the variations in the relationship between Hcy and δNfL based on race and sex. This was done by incorporating Hcy × *TIME* × Race and Hcy × *TIME* × sex into the analysis.

The two-stage Heckman selection technique was applied to all model by including an inverse mills ratio in the mixed-effect regression models, as described elsewhere [39, 50]. Two additional analyses were conducted using two other alternative measures of Hcy: **(A)** Time-dependent Hcy (Log_e_ transformed) on multiple-imputed data for time-dependent covariates (Hcy_td_); **(B)** Hcy (Log_e_ transformed) trajectory group (e.g. “High vs. Low”; “Medium vs. Low”) based on GBTM. A similar modeling strategy was employed for both secondary analyses **(A)** and **(B)**. A sensitivity analysis was also conducted by excluding non-fasting participants.

We visualized our findings by utilizing predictive margins along with their estimated 95% CI. Specifically, longitudinal plasma NfL outcome was plotted across time and by Hcy exposure levels. This was done mainly for the overall sample, while basing these estimations on results from the main mixed-effects linear regression models. A priori, main effects and interactions had predetermined type I error rates set at 0.05 and 0.10, respectively [51].

## Results

### Sex-stratified descriptive findings

**Fig. 2:**
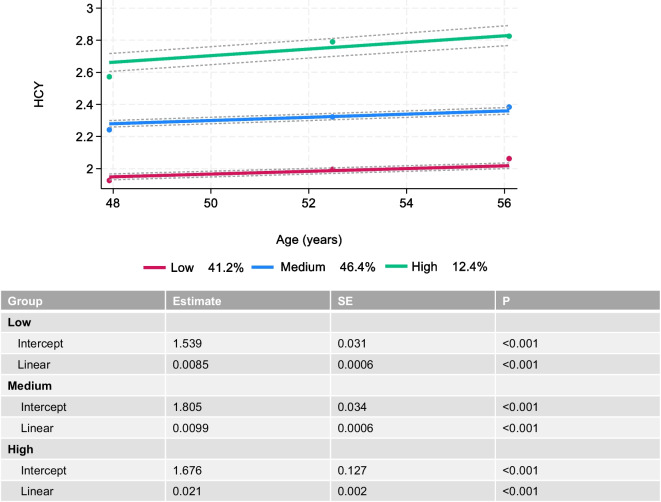
Group-based trajectory model findings for 3 groups of Hcy (Log_e_ transformed): HANDLS 2004–2017. Abbreviations: HANDLS = Healthy Aging in Neighborhoods of Diversity Across the Life Span; HCY = Log_e_ transformed blood homocysteine

Among the 690 HANDLS participants, there were 399 females and 291 men. The sample featured 308 White adults, and 382 African American adults. Figure 2 presents the results of the GBTM which identified three distinct groups for Hcy trajectories, labelled based on intercept and slope as: “Low increasing”; “Medium increasing” and “High increasing”. Detailed results, including predicted probabilities of group inclusion, are shown in **Table S2**, along with results from the linear models with 1 and 2 group solutions, showcasing greater BIC compared to the 3-group solution.

We observed sex differences in several covariates as illustrated in Table 1. For example, females were less likely than their male counterparts to be “current illicit drug users” (11% vs. 24%), more likely to be obese (54.9% vs. 31.9%), having higher rates of self-reported cardiovascular disease (15.6% vs. 8.7%). Females also had higher CES-D mean scores, lower energy intake, and lower prevalence of pre-diabetes. Hcy was consistently higher among male participants compared to females, with the “low increasing” group being significantly less prevalent among males (26.8% vs. 53.9%). Similarly, NfL at v_1_ and v_3_ were more elevated among male participants, while the observed rate of change in NfL did not differ between male and female participants.

**Table 1:** Study sample characteristics by sex and by race: HANDLS, 2004–2017^1^

	Overall (*n* = 690)	Female (*n* = 399)	Male (*n* = 291)	*P*_sex_	White (*n* = 308)	African American (*n* = 382)	*P*_race_
**Socio-demographic, lifestyle and health-related factors at v**_**1**_							
***Sex, %***				…			0.65
male	42.1	…	100.0		41.2	42.9	
female	57.9	100.0	…		58.8	57.1	
***Race, %***				0.65			…
African American	55.4	54.6	56.4		…	100.0	_
White	44.6	45.5	43.6		100.0	…	
Age, years	47.8 ± 0.3	48.0 ± 0.5	47.6 ± 0.5	0.49	48.5 ± 0.5	47.3 ± 0.5	0.071
***Poverty status, %***				0.27			0.46
Below Poverty	28.7	30.3	26.5		27.3	29.8	
Above Poverty	71.3	69.7	73.5		72.7	70.2	
***Education, %***							
< High School	5.5	5.0	5.9	0.78	8.6	2.9	0.004^5^
High School	57.2	56.2	58.6	Ref	57.2	57.2	Ref
> High School	37.3	38.6	35.5	0.45	34.2	39.8	0.35
Current illicit drug use, % yes	16.5	10.9	24.1	< 0.001^5^	13.1	19.3	0.034
Current tobacco use, % yes	39.6	39.7	46.5	0.082	42.1	43.0	0.80
Healthy Eating index, HEI-2010 total score	42.1 ± 0.5	42.7 ± 0.7	41.2 ± 0.8	0.16	41.3 ± 0.8	42.7 ± 0.6	0.22
Energy intake, kcal/d	1983 ± 43	1676 ± 42	2405 ± 81	< 0.001^5^	1983 ± 60	1983 ± 59	0.99
CES-D total score	14.2 ± 0.4	14.7 ± 0.6	13.5 ± 0.6	0.17	15.2 ± 0.7	13.4 ± 0.6	0.035^5^
**Body mass index at v1, BMI**_**v1**_**,kg.m**^**−2**^	30.2 ± 0.3	31.7 ± 0.4	31.7 ± 0.4	< 0.001^5^	30.2 ± 0.4	30.2 ± 0.4	0.97
**Weight status at v**_**1**_**, %**							
Underwt.: BMI_v1_ < 18.5 kg.m^−2^	2.6	2.5	2.7	0.20	2.6	2.6	0.71
Normal: BMI_v1_ ≥ 18.5 and < 25	22.0	17.3	28.6	< 0.001^5^	22.4	21.7	0.94
Overwt.: BMI_v1_ ≥ 25 and < 30	30.1	25.3	36.8	< 0.001^5^	30.8	29.6	0.64
Obese: BMI_v1_ ≥ 30 kg.m^−2^	45.2	54.9	31.9	Ref	44.2	46.1	Ref
**Allostatic load total score at v**_**1**_**, AL**_**total**_	1.84 ± 0.05	1.88 ± 0.06	1.80 ± 0.08	0.34	1.92 ± 0.07	1.78 ± 0.06	0.13
Hypertension, % yes	41.3	43.3	38.6	0.23	35.7	45.9	0.008^5^
Diabetes, %							
No	68.2	70.7	64.7	Ref	64.5	71.2	Ref
Pre-diabetes	19.9	16.6	24.5	0.017^5^	22.8	17.6	0.071
Diabetes	11.9	12.6	10.8	0.78	12.7	11.2	0.35
Hypercholesterolemia, % yes	24.8	24.6	25.1	0.90	30.3	20.5	0.004^5^
Cardiovascular disease^2^, % yes	12.7	15.6	8.7	0.011^5^	11.0	14.1	0.23
**Plasma Homocysteine concentration, Log**_**e**_** transformed**							
Hcy_v1_	2.144 ± 0.013	2.057 ± 0.015	2.265 ± 0.021	< 0.001^5^	2.137 ± 0.017	2.151 ± 0.019	0.59
Hcy_v2_	2.229 ± 0.014	2.139 ± 0.015	2.353 ± 0.024	< 0.001^5^	2.216 ± 0.017	2.240 ± 0.021	0.38
Hcy_v3_	2.291 ± 0.013	2.229 ± 0.017	2.377 ± 0.019	< 0.001^5^	2.266 ± 0.018	2.311 ± 0.018	0.080^5^
Hcy_traj_^3^							
Low increasing	42.5	53.9	26.8	< 0.001^5^	45.5	40.1	0.42
Medium increasing	46.2	38.6	56.7	Ref	46.1	46.3	Ref
High increasing	11.3	7.5	16.5	0.12	8.4	13.6	0.075
**Plasma NfL, Log**_**e**_** transformed**							
NfL_v1_	1.98 ± 0.02	1.95 ± 0.02	2.02 ± 0.03	0.046^5^	2.09 ± 0.03	1.89 ± 0.03	< 0.001^5^
NfL_v3_	2.35 ± 0.02	2.30 ± 0.03	2.41 ± 0.03	0.016^5^	2.40 ± 0.03	2.30 ± 0.03	0.020
δNfL_obs_^4^	0.0482 ± 0.0026	0.0484 ± 0.0031	0.0481 ± 0.0045	0.94	0.0470 ± 0.0041	0.0493 ± 0.0033	0.66

AL_total_ = Allostatic load total score; BMI = Body mass index; bayes = Empirical bayes estimator; CES-D = Center for Epidemiologic Studies-Depression; δ = Annualized rate of change; HANDLS = Healthy Aging in Neighborhoods of Diversity Across the Life Span; NfL = plasma Neurofilament Light; Overwt. = Overweight; Underwt. = underweight; v_1_ = Visit 1; v_2_ = Visit 2; v_3_ = Visit 3

^1^ Values are means ± SE for continuous variables or % for categorical variables

^2^ cardiovascular disease status is based on several self-reported conditions namely angina, atrial fibrillation, coronary artery disease, myocardial infarction and congestive heart failure

^3^ Hcy_traj_ is the grouping of time-dependent Hcy obtained from group-based trajectory models with age as the time variable

^4^ Observed annual rate of change in NfL between v_1_ and v_3_, validated against the empirical bayes estimator predicted from mixed-effects linear regression model with NfL as outcome and *TIME* as the only predictor (Pearson’s r > 0.80). All SD can be obtained from this Table as sqrt(N) × SE to assess clinically meaningful effects

^5^*P* < 0.05 upon further adjustment for age, sex, race and poverty status in multiple linear and multinomial logit models

African American participants had lower SES in terms of poverty status and educational attainment, were more likely to screen positive for hypertension, but less likely to screen positive for hypercholesterolemia. While no difference by Hcy exposure was noted, NfL at v_1_ and v_3_ were lower among African American participant compared with their White counterparts.

### Plasma Hcy concentrations (initial visit, time-dependent and trajectories) and their longitudinal association with plasma NfL

**Fig. 3:**
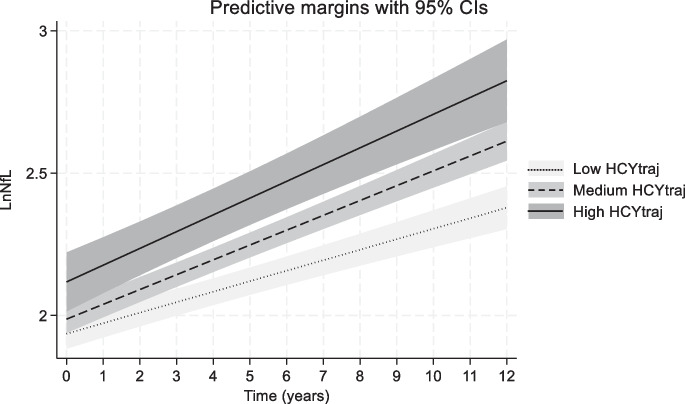
Predictive margins of LnNfL over follow-time vs. group-based trajectories of LnHcy: HANDLS 2004–2017. Abbreviations: HANDLS = Healthy Aging In Neighborhoods of Diversity Across the Life Span; Hcy_traj_ = trajectory groups of LnHcy obtained using group-based trajectory models; Ln = Loge transformed; NfL = plasma Neurofilament Light; v_1_ = Visit 1; v_2_ = Visit 2; v_3_ = Visit 3. Note: Based on fully adjusted mixed-effects linear regression model with main exposure being Hcyt_raj_ and outcome being LnNfL. Model is adjusted for age, sex, race, poverty status, education, current smoking status, current drug use status, HEI-2010, energy intake, body mass index, allostatic load, hypertension, diabetes, dyslipidemia, cardiovascular disease and the total CES-D score, measured at v_1_. Predictive margins obtained from mixed-effects linear regression model conducted on the first imputation. All other imputation gave a similar pattern of association

Table 2 outlines the results from mixed-effects linear regression models assessing the relationship between Hcy exposures and NfL between v_1_ and v_3_ (2004–2017). In **Model A**, v_1_ Hcy was used as the main exposure and interacted with TIME to assess its association at baseline and longitudinal annual rate of change in NfL. In the unadjusted and reduced models ***(Model 0.A.*** and ***Model 1. A.***) as well as in the fully adjusted model (***Model 2. A.***), v_1_ Hcy was positively associated with v_1_ NfL (Fully adjusted: γ_0v1_ =  + 0.133 ± 0.031, P < 0.001). However, v_1_ Hcy was not associated with annualized rate of change in NfL based on findings in models that adjusted for other potentially confounding covariates. In contrast, when time-dependent Hcy (Hcy_td_) was considered along with time-dependent covariates on multiple-imputed data (**Model B**), both v_1_ NfL and annualized rate of change in NfL were associated with Hcy_td_, with a significant sex difference when it came to the longitudinal association (Hcy_td_ vs. δNfL). Specifically, in unadjusted (***Model 0.B***), reduced (***Model 1.B.***) and fully adjusted (***Model 2.B.***) models, among males only, Hcy_td_ was associated with faster increase in NfL over time (fully adjusted: γ_1td_ =  + 0.022 ± 0.007, P < 0.010 among male participants, P < 0.10 for Hcy_td_ × *TIME* × Sex in separate unstratified fully adjusted model). No sex or racial differences were noted in the association between Hcy_td_ and v_1_ NfL (overall, fully adjusted: γ_1td_ =  + 0.126 ± 0.025, P < 0.001). Without any notable sex or racial differences, the GBTM Hcy exposure indicating that individuals belonging to the “High increasing” group when compared with the “Low increasing group” were consistently associated with both higher v1 NfL and faster increase in NfL (overall, fully adjusted, High *vs*. Low: γ_0gbtm_ =  + 0.226 ± 0.058, P < 0.001; γ_1gbtm_ =  + 0.022 ± 0.007, P < 0.010). In addition, a dose–response relationship was observed in both the cross-sectional and longitudinal models whereby the strength of the association between High vs. Low was greater than the strength of association between Medium vs. Low. The GBTM exposure findings are further illustrated using predictive margins from the fully adjusted model (Model 2.C., Fig. 3).

**Table 2:** Plasma homocysteine (Hcy) at initial visit (Hcy_v1_), time-dependent Hcy (Hcy_td_) and trajectories in plasma Hcy (Hcy_traj_) and their association with baseline and annualized change in plasma NfL between v_1_ and v_3_, overall and by sex and race: mixed-effects linear regression models; HANDLS, 2004–2017^1^

	Overall (*n* = 690)	Female (*n* = 399)	Male (*n* = 291)	*P*_sex_^2^	White (*n* = 308)	African American (*n* = 382)	*P*_race_^3^
**Hcy**_**v1**_	*						
Model 0.A., per SD LnHcy							
Hcy_v1_,* γ*_*0a*_	+ 0.219 ± 0.034***	+ 0.237 ± 0.049***	+ 0.194 ± 0.052***	0.53	+ 0.187 ± 0.052***	+ 0.242 ± 0.044***	0.44
Hcy_v1_ × *TIME*, *γ*_*1a*_	+ 0.008 ± 0.004*	+ 0.004 ± 0.005	+ 0.012 ± 0.006	0.27	+ 0.014 ± 0.007*	+ 0.005 ± 0.005	0.27
Model 1.A., per SD LnHcy							
Hcy_v1_,* γ*_*0a*_	+ 0.139 ± 0.032***	+ 0.101 ± 0.043*	+ 0.170 ± 0.048***	0.23	+ 0.151 ± 0.049**	+ 0.125 ± 0.042**	0.74
Hcy_v1_ × *TIME*, *γ*_*1a*_	0.006 ± 0.0004	+ 0.000 ± 0.006	+ 0.010 ± 0.006	0.28	+ 0.013 ± 0.007	+ 0.003 ± 0.005	0.36
Model 2.A., per SD LnHcy							
Hcy_v1_,* γ*_*0a*_	+ 0.133 ± 0.031***	+ 0.091 ± 0.041*	+ 0.162 ± 0.048**	0.24	+ 0.131 ± 0.048**	+ 0.121 ± 0.042**	0.75
Hcy_v1_ × *TIME*, *γ*_*1a*_	+ 0.006 ± 0.004	−0.000 ± 0.006	+ 0.010 ± 0.006	0.23	+ 0.013 ± 0.007	+ 0.003 ± 0.005	0.45
**Hcy**_**td**_							
Model 0.B., per SD LnHcy							
Hcy_td_,* γ*_*0a*_	+ 0.162 ± 0.026***	+ 0.162 ± 0.035***	+ 0.146 ± 0.041***	0.64	+ 0.141 ± 0.041**	+ 0.177 ± 0.033***	0.44
Hcy_td_ × *TIME*, *γ*_*1a*_	+ 0.016 ± 0.004***	+ 0.007 ± 0.005	+ 0.027 ± 0.007***	**0.023**^***2***^	+ 0.019 ± 0.007**	+ 0.014 ± 0.005**	0.45
Model 1.B., per SD LnHcy							
Hcy_td_,* γ*_*0a*_	+ 0.137 ± 0.026***	+ 0.128 ± 0.035***	+ 0.143 ± 0.040***	0.54	+ 0.131 ± 0.041**	+ 0.141 ± 0.034***	0.53
Hcy_td_ × *TIME*, *γ*_*1a*_	+ 0.016 ± 0.004***	+ 0.006 ± 0.006	+ 0.026 ± 0.007***	***0.050***^***2***^	+ 0.018 ± 0.07*	+ 0.015 ± 0.005**	0.59
Model 2.B., per SD LnHcy							
Hcy_td_,* γ*_*0a*_	+ 0.126 ± 0.025***	+ 0.114 ± 0.035**	+ 0.133 ± 0.039**	0.44	+ 0.115 ± 0.041**	+ 0.134 ± 0.034***	0.55
Hcy_td_ × *TIME*, *γ*_*1a*_	+ 0.015 ± 0.004***	+ 0.005 ± 0.006	+ 0.022 ± 0.007**	***0.075***^***2***^	+ 0.017 ± 0.007**	+ 0.014 ± 0.005**	0.79
**Hcy**_**traj**_							
Model 0.C., Hcy_traj_^4^							
[Medium. vs. Low],* γ*_*0a*_	+ 0.0045 ± 0.0417	+ 0.0400 ± 0.0520	−0.0951 ± 0.0736	0.13	−0.0312 ± 0.055	+ 0.0489 ± 0.0593	0.33
[High vs. Low],* γ*_*0a*_	+ 0.168 ± 0.065*	+ 0.0473 ± 0.0957	+ 0.1663 ± 0.0982	0.39	+ 0.1774 ± 0.0983	+ 0.2141 ± 0.0862*	0.80
[Medium. vs. Low] × *TIME*, *γ*_*1a*_	+ 0.0140 ± 0.0046**	+ 0.0149 ± 0.0056**	+ 0.0142 ± 0.0085	0.97	+ 0.0141 ± 0.0072	+ 0.0130 ± 0.0061*	0.91
[High vs. Low] × *TIME*, *γ*_*1a*_	+ 0.021 ± 0.07**	+ 0.02012 ± 0.0101*	+ 0.0232 ± 0.0112*	0.77	+ 0.0266 ± 0.0129*	+ 0.0185 ± 0.0087*	0.66
Model 1.C., Hcy_traj_^4^							
[Medium. vs. Low],* γ*_*0a*_	+ 0.045 ± 0.037	+ 0.086 ± 0.043*	−0.003 ± 0.067	0.33	+ 0.054 ± 0.051	+ 0.030 ± 0.053	0.87
[High vs. Low],* γ*_*0a*_	+ 0.243 ± 0.057***	+ 0.131 ± 0.079	+ 0.291 ± 0.090**	***0.087***^***2***^	+ 0.285 ± 0.090**	+ 0.214 ± 0.077**	0.95
[Medium. vs. Low] × *TIME*, *γ*_*1a*_	+ 0.015 ± 0.005**	+ 0.015 ± 0.006**	+ 0.015 ± 0.009	1.00	+ 0.016 ± 0.007*	+ 0.014 ± 0.006*	0.98
[High vs. Low] × *TIME*, *γ*_*1a*_	+ 0.023 ± 0.007**	+ 0.022 ± 0.010*	+ 0.022 ± 0.012	0.95	+ 0.028 ± 0.013*	+ 0.022 ± 0.009*	0.81
Model 2.C., Hcy_traj_^4^							
[Medium. vs. Low],* γ*_*0a*_	+ 0.050 ± 0.037	+ 0.083 ± 0.042*	+ 0.012 ± 0.067	0.25	+ 0.047 ± 0.051	+ 0.033 ± 0.054	1.00
[High vs. Low],* γ*_*0a*_	+ 0.226 ± 0.058***	+ 0.127 ± 0.077	+ 0.290 ± 0.093	0.19	+ 0.262 ± 0.092**	+ 0.193 ± 0.079*	0.84
[Medium. vs. Low] × *TIME*, *γ*_*1a*_	+ 0.015 ± 0.0048**	+ 0.015 ± 0.006**	+ 0.014 ± 0.009	0.91	+ 0.014 ± 0.007*	+ 0.016 ± 0.006*	0.67
[High vs. Low] × *TIME*, *γ*_*1a*_	+ 0.022 ± 0.007**	+ 0.020 ± 0.010	+ 0.019 ± 0.012	0.69	+ 0.027 ± 0.013*	+ 0.022 ± 0.009*	0.90

BMI = Body mass index; CES-D = Center for Epidemiologic Studies-Depression; δ = Annualized rate of change; HANDLS = Healthy Aging in Neighborhoods of Diversity Across the Life Span; HEI = Healthy Eating Index; NfL = plasma Neurofilament Light; v_1_ = Visit 1; v_2_ = Visit 2; v_3_ = Visit 3

^1^ Values are fixed effects γ ± SE. Models A-C included each of v_1_ Hcy (Loge transformed), Hcy_td_ and Hcy_traj_, separately as the main predictor for v1 NfL and NfL annualized change over time (δNfL), using a series of mixed-effects linear regression models, carried out in the overall population, and stratified by sex and by race, separately. Model 0 did not adjust for any other covariate. Model 1 adjusted only for age, sex, race, poverty status, educational attainment and the inverse mills ratio. Model 2 followed a similar approach but adjusted further for selected lifestyle and health-related factors, namely current drug use, current tobacco use, HEI-2010, total energy intake, BMI, the allostatic load total score, self-reported hypertension and diabetes, measured hypercholesterolemia and statin use, self-reported cardiovascular disease and the CES-D total score. *a* subscript can be either of 3 exposures: v_1_; td; gbtm (or Hcy_traj_)

^2^ *P*_sex_ are based on separate models testing the statistical significance for Sex × Hcy and Sex × Hcy × TIME in models that are unstratified by sex or race to which these 2-way and 3-way interaction terms were included for each socio-demographic factor, separately

^3^ *P*_race_ are based on separate models testing the statistical significance for Race × Hcy and Race × Hcy × TIME in models that are unstratified by sex or race to which these 2-way and 3-way interaction terms were included for each socio-demographic factor, separately

^4^ “Low” category for Hcy_traj_ had a prevalence of 41.2%, “Medium” category had a prevalence of 46.4%, while the “High” category had a prevalence of 12.4%. See Fig. 2 for ranges of values over time (age in years). Values need to be exponentiated to obtain the raw values of Hcy

^*^P < 0.05; **P < 0.010; ***P < 0.001 for null hypothesis that fixed effect γ = 0

## Discussion

### Summary of findings

This study investigated the longitudinal associations of initial-visit plasma Hcy concentration with initial visit NfL (NfL_v1_) and longitudinal change in NfL (δNfL)over time, independently of key exogenous confounders, and across sex and race. The study also assessed whether time-dependent values and trajectories of plasma Hcy concentrations over time were associated with these two outcomes of interest. In Model A, Hcy_v1_ was positively associated with NfL_v1_, but not with annualized rate of change in NfL. However, when time-dependent Hcy (Hcy_td_) was considered along with time-dependent covariates on multiple-imputed data, both NfL_v1_ and annualized rate of change in NfL were associated with Hcy_td_, with significant sex difference in the longitudinal association. The GBTM Hcy exposure indicated that individuals belonging to the "High increasing" group were consistently associated with both higher NfL_v1_ and faster increase in NfL.

#### Previous studies

### Plasma NfL and its relationship with neurocognitive outcomes and mortality

Accumulating evidence indicates that plasma NfL may be a useful blood-based biomarker for neurodegeneration [52]. Acquiring blood is much less invasive than CSF, giving blood-based NfL measurements an advantage for biomarker utilization [52]. However, plasma NfL is notably a non-specific marker of neuronal injury and damage, which may be caused by a multitude of factors and disease processes including ischemia and inflammation[52]. Importantly, elevated plasma NfL levels have also been recently linked to mortality[53–55]. Therefore, it is important to further understand the relationship between plasma NfL and other blood-based markers, such as Hcy, to decipher molecular mechanisms that contribute to neurodegenerative disorders, diseases and adverse health outcomes.

### Plasma Hcy and its association with neuro-cognitive outcomes

Elevated blood levels of homocysteine (Hcy) pose a risk for a range of dementia spectrum illnesses, such as AD, vascular dementia, frontotemporal dementia, and Lewy body dementia [56]. Vitamin B12 and folate are essential cofactors for the process of Hcy methylation [56]. Deficiency in either vitamin B-12 or folate can lead to elevated levels of Hcy [56]. Moreover, consuming vitamins B-6 and B-12 was associated with larger brain size, specifically in the hippocampus and amygdala regions, and reduced severity of white matter lesions [57, 58]. A comprehensive randomized controlled study called VITACOG uncovered positive links between high doses of B vitamins and regions of the brain that are susceptible to AD. This was achieved by reducing the tissue atrophy rate within these regions over a period of two years [59]. The trial demonstrated that supplementing with B vitamins can help maintain executive function and prevent decline in overall cognition, as well as in episodic and semantic memory [60]. Nevertheless, a recent meta-analysis of 23 randomized controlled trials revealed that there were no notable disparities in cognitive function scores between the groups that received supplementation and those that received a placebo. This indicates that supplementation did not yield any advantages over placebo in terms of preventing or decelerating the decline in cognitive function [61].

### Plasma Hcy and its association with plasma NfL and other markers of neurodegeneration

One study reported a strong correlation between CSF NfL and plasma homocysteine levels in 83 HIV-1-positive individuals. Vitamin B-12 and folate levels were inversely correlated with homocysteine, suggesting among others, that brain damage may be related to functional cobalamin/folate insufficiency[26]. Researchers looking at the relationships between neurodegenerative biomarkers, inflammation, and nutrition in older community-dwelling persons (70 + , n = 475, Multidomain Alzheimer Preventive Trial (MAPT)) discovered that progranulin and plasma neurofilament light chain are related to TNFR-1 and GDF-15 [27]. The findings specifically demonstrated that NfL was positively correlated with GDF-15, TNFR-1, IL-6, and Hcy, while TNFR-1 was not related to nutritional or inflammatory indicators [27]. Progranulin showed a positive correlation with MCP-1, TNFR-1, and GDF-15[27]. These findings corroborate earlier research showing a connection between inflammatory pathways and neurodegenerative plasma markers [27]. An earlier animal model study found that protein phosphatase PP2A in the rat brain dephosphorylates neurofilaments and tau, potentially linking methylation and neurodegeneration[28]. Elevated S-adenosylhomocysteine (SAH) and low S-adenosylmethionine (SAM) may increase dementia risk [28]. Hyperhomocysteinemia affects P-tau, pNF-H, and PP2A levels and activity in the brain. Plasma folate may prevent neurodegeneration, as it correlates with these findings [28]. Finally, in recent randomized controlled trials for B-vitamin supplementation, there was a strong association between levels of plasma NfL and plasma Hcy [29]. The group receiving the active treatment experienced a 35% reduction in plasma Hcy levels from the beginning to the end of the 12-month period [29]. Nevertheless, there were no notable alterations found in the amounts of NfL in the plasma or in cognitive assessments [29]. The study determined that the association between neurofilament light protein and homocysteine was not influenced by vitamin B, requiring further investigation into causal associations [29].

#### Strengths and limitations

Among its many strengths, our study had a sufficient sample size ascribed to African American adults to perform subset analyses, particularly with respect to potential risk factors for cognitive impairment and dementia in this group. Second, this work is the first to investigate the correlation between blood homocysteine and plasma NfL, addressing crucial research problems. Our findings implied that it is crucial to account for blood Hcy when evaluating the practicality of using plasma NfL as a predictor of neurocognitive outcomes. Furthermore, plasma NfL may serve as a mediating mechanism through which Hcy can impact neurocognitive outcomes. Third, we determined the timing of the relationship using a longitudinal study design that analyzed initial exposures and their impact on changes in outcomes over time, thus ascertaining temporality of these relationships. Furthermore, this study employed sophisticated statistical methods such as multiple linear mixed-effects regression (both for initial time-point exposure and time-dependent concurrent exposures), and GBTM to examine the relationships and variations across sex and race. The analysis also accounted for important factors that could influence the results and addressed potential biases in the sample using 2-stage Heckman selection.

However, our investigation has several notable limitations. First, a lower baseline plasma NfL compared to previous studies had more older adults in their samples make our findings less comparable to prior investigations. Specifically, both baseline and the rate of change in NfL may have been slower in our study. Multiple studies have shown that the likelihood of experiencing negative cognitive effects in the future is influenced by factors related to cardiovascular and metabolic health, as well as lifestyle choices such as diet which may influence blood Hcy, throughout middle age [62, 63]. Thus, our research demonstrates that a potential indicator of neurodegeneration in midlife is indeed linked over time to the blood Hcy levels in middle-aged individuals. Our analysis relied on self-reported data for some lifestyle factors, which can introduce recall bias and affect the accuracy of the results. Furthermore, due to the limited available and detailed information on specific medications, we were not able to control for the use of phenytoin which is associated with elevated Hcy [64]. Furthermore, chronic kidney disease as measured by estimated glomerular filtration rate or eGFR (a measure that relies on plasma creatinine levels) or cystatin C may also be associated both with Hcy and NfL[65, 66]. However, the strong correlation between Hcy and creatinine based on national data [67], coupled with the ambiguity of the causal chain of events render them potential mediators between Hcy and NfL. Therefore, it is important in the future, to study the longitudinal and dynamic associations of Hcy, CKD and NfL or other neurodegenerative markers. Additionally, our study population is not fully representative of the general population, limiting the generalizability of our findings. Finally, while we accounted for many confounding variables, there is always the possibility of residual confounding that could impact the observed associations.

Dynamic Hcy exposures (Hcy_td_ and Hcy_gbtm_) were associated with faster rate of increase in NfL over time, reflecting potentially faster rate of axonal degeneration. It is also essential to explore the potential mechanisms underlying the observed associations, which may provide insights into therapeutic targets for preventing or slowing neurodegeneration. Additionally, future research should consider a more diverse population sample and incorporate longitudinal measures of other biomarkers related to neurodegeneration. Understanding the interplay between Hcy, NfL, and other risk factors across different populations can help in developing targeted prevention strategies. Further studies are needed in comparable populations to replicate our findings.

## Supplementary Information

Below is the link to the electronic supplementary material.

Supplementary file1 (DOCX 333 KB)

## Data Availability

Upon request, data can be made available to researchers with approved proposals, after they have agreed to confidentiality as required by our IRB. Policies are publicized on: https://handls.nih.gov. Data access request can be sent to principal investigators (PI) or the study manager, Jennifer Norbeck at norbeckje@mail.nih.gov. These data are owned by the National Institute on Aging at the NIH. The PIs have made those data restricted to the public for two main reasons: “(1) The study collects medical, psychological, cognitive, and psychosocial information on racial and poverty differences that could be misconstrued or willfully manipulated to promote racial discrimination; and (2) Although the sample is fairly large, there are sufficient identifiers that the PIs cannot guarantee absolute confidentiality for every participant as we have stated in acquiring our confidentiality certificate.” Code book and statistical analysis script can be readily obtained from the corresponding author, upon request, by e-mail contact at baydounm@mail.nih.gov.
