# Supplementary material for: Plasma homocysteine level and trajectories in association with longitudinal increase in plasma neurofilament light among urban adults
Source: GeroScience. 2025 Feb 19;47(3):4663–78. doi: 10.1007/s11357-025-01567-z (PMC12181452; doi:10.1007/s11357-025-01567-z)
Supplement: Supplementary file 1 — Supplementary file1 (DOCX 333 KB) [file 11357_2025_1567_MOESM1_ESM.docx]

**APPENDICES**

**Supplementary Method 1: NfL sample selection**

Plasma NfL levels were measured in a subset of people from the HANDLS project during visits v_1_ (2004-2009), v_2_ (2009-2013), and v_3_ (2013-2018). For our current investigation, we only used data from visits v_1_ and v_2_. The sub-sample consisted of 238 participants from the HANDLS SCAN, which is an additional study focused on neuroimaging. (1) This particular analysis within the HANDLS cohort removed individuals who had a previous diagnosis of dementia, stroke, transient ischemic attack, or carotid endarterectomy. It also eliminated those who had contraindications for MRI, terminal illness, HIV infection, or other neurological illnesses. (1) All participants in the HANDLS SCAN sub-study provided plasma samples at three visits, except for one individual who only provided samples at two visits. Furthermore, our study encompassed a total of 463 participants, with a combined total of 1,389 plasma samples collected at three different time points (v_1_, v_2_, and v_3_). These participants were specifically selected based on the following criteria: they tested negative for HIV, completed cognitive tests (specifically the Trailmaking test, part A and Digits Span-Forward) at v1 and v2, provided Centers of Epidemiologic Studies-Depression scores at all three visits, and had no prior medical history of HIV, stroke, transient ischemic attack, dementia, epilepsy, Parkinson's disease, or brain cancer. Three participants were included in the study who had plasma samples available from v1, v2, and v3. These participants also had genome-wide DNA methylation data at v1(2, 3, 4). Participants were excluded based on the criteria mentioned before. Therefore, a total of 694 persons from the HANDLS study had plasma NfL data at v1, while 709 participants had data at v2, and 707 participants had data at both v1 and v2.

**Supplementary Table 1.** Allostatic load indicator criteria (5).

|  | **High-risk clinical** |
| --- | --- |
| Waist:Hip ratio | >0.9 for men; > 0.85 for women (6) |
| Albumin (g/dL) | < 3.8 (7) |
| C-reactive protein (mg/dL) | ≥ 0.3 (8) |
| Total cholesterol (mg/dL) | ≥240(9) |
| HDL (mg/dL) | <40(9) |
| Glycated hemoglobin (%) | ≥6.4(10, 11) |
| Resting heart rate (beat/min) | ≥90(12) |
| Systolic BP | ≥140(13) |
| Diastolic BP | ≥90(13) |

**Supplementary method 2**: Mixed-effects regression models

The primary multiple mixed-effects regression models can be stated as follows:

**Multi-level models** vs. **Composite models**

| **Eq.**  **1.1-1.4** |  |  |  |
| --- | --- | --- | --- |

The variable Yij represents the outcome, which is the measurement of plasma NfL at visits v_1_, v_2_, and/or v_3_ for each individual "i" and visit "j". The term represents the individual-level intercept, represents the individual-level slope, represents the level-2 intercept of the random intercept , represents the level-2 intercept of the slope , and represents a vector of fixed covariates for each individual "i" that are used to predict the level-1 intercepts and slopes, which may include socio-demographic variables and others. The study incorporated mixed-effects regression models that included the Hcy exposure measured at v1 (X_ij_) in the primary part of the analysis, as well as covariates (Z_ij_). Both and are level-2 disturbances, while is the within-person level-1 disturbance(14).

It is important to mention that the models were trained on the entire HANDLS cohort, which had complete data on either v_1_, v_2_, or v_3_ of NfL. This was done to enhance the accuracy and dependability of the predicted estimations. Predictions of empirical Bayes estimators for the annual rate of change in NfL (δNfL) were derived from time-interval mixed-effects models. The outcome variable was plasma NfL, with up to 3 repeats and the only predictor was *TIME*. This estimate was utilized as a means of validating the observed annualized change in NfL between the three visits. The latter was calculated as the average of the annualized changes of Log_e_ converted NfL between v_1_ and v_2_, v_2_ and v_3_, and v_1_ and v_3_. The observed annualized rate of change at the individual level (δNfLobs) was determined by 1, 2, or 3 values of annualized changes. Therefore, there were no further missing data for this estimate. The scatter plot displaying the relationship between δNfLobs and δNfLbayes may be observed in **Supplementary Figure 1**.

**Supplementary Figure 1.** Observed vs. empirical bayes estimator for annualized rate of change in Log_e_ transformed NfL, Pearson’s r=0.76, p<0.001

*P<0.05; **P<0.010; ***P<0.001 for null hypothesis that path coefficient α=0.

**Supplementary Table 2**. Results of group-based trajectory model for time-dependent blood homocysteine in waves 1, 3 and 4 of HANDLS: 2004-2017

| . traj if sampleHCY==1, var(Lnw1HCys Lnw3HCys Lnw4HCys) indep(w1Age w3Age w4Age) model(cnorm) max1(400) order(1 1 1) sigmabygroup detail | | | | | | | | | | | | | |
| --- | --- | --- | --- | --- | --- | --- | --- | --- | --- | --- | --- | --- | --- |
|  |  |  |  |  |  |  |  |  |  |  |  |  |  |
| ==== traj stata plugin ==== Jones BL Nagin DS, build: May 2 2023 | | | | | | |  |  |  |  |  |  |  |
|  |  |  |  |  |  |  |  |  |  |  |  |  |  |
| 3720 observations read. | | |  |  |  |  |  |  |  |  |  |  |  |
| 2144 excluded by if condition. | | |  |  |  |  |  |  |  |  |  |  |  |
| 1576 observations used in the trajectory model. | | | | |  |  |  |  |  |  |  |  |  |
|  |  |  |  |  |  |  |  |  |  |  |  |  |  |
| Start |  |  |  |  |  |  |  |  |  |  |  |  |  |
| Parameter estimates | |  |  |  |  |  |  |  |  |  |  |  |  |
|  |  |  |  |  |  |  |  |  |  |  |  |  |  |
| 1.87835, 0.00000, 2.22918, 0.00000, 2.58002, 0.00000, | | | | | | |  |  |  |  |  |  |  |
| 0.35084, 0.35084, 0.35084, 33.33333, 33.33333, 33.33333 | | | | | | |  |  |  |  |  |  |  |
|  |  |  |  |  |  |  |  |  |  |  |  |  |  |
|  |  |  |  |  |  |  |  |  |  |  |  |  |  |
| Neg. Log Percent | | |  |  |  |  |  |  |  |  |  |  |  |
| Likelihood Decrease | | |  |  |  |  |  |  |  |  |  |  |  |
|  |  |  |  |  |  |  |  |  |  |  |  |  |  |
|  |  |  |  |  |  |  |  |  |  |  |  |  |  |
| 0 1476.3983079 | | |  |  |  |  |  |  |  |  |  |  |  |
| too big of a step | |  |  |  |  |  |  |  |  |  |  |  |  |
| 1 1432.7681320 2.95517651 | | | |  |  |  |  |  |  |  |  |  |  |
| 2 1364.8685806 4.73904673 | | | |  |  |  |  |  |  |  |  |  |  |
| 3 1363.3517463 0.11113409 | | | |  |  |  |  |  |  |  |  |  |  |
| 4 1352.2135101 0.81697451 | | | |  |  |  |  |  |  |  |  |  |  |
| 5 1349.5384467 0.19782847 | | | |  |  |  |  |  |  |  |  |  |  |
| 6 1341.1179063 0.62395707 | | | |  |  |  |  |  |  |  |  |  |  |
| 7 1340.3080011 0.06039030 | | | |  |  |  |  |  |  |  |  |  |  |
| 8 1340.0883039 0.01639155 | | | |  |  |  |  |  |  |  |  |  |  |
| 9 1339.6706339 0.03116735 | | | |  |  |  |  |  |  |  |  |  |  |
| 10 1339.2995650 0.02769852 | | | |  |  |  |  |  |  |  |  |  |  |
| 11 1210.5671292 9.61192247 | | | |  |  |  |  |  |  |  |  |  |  |
| 12 1038.9013759 14.18060586 | | | |  |  |  |  |  |  |  |  |  |  |
| 13 1002.1501035 3.53751311 | | | |  |  |  |  |  |  |  |  |  |  |
| 14 948.3632296 5.36714747 | | | |  |  |  |  |  |  |  |  |  |  |
| 15 918.9742628 3.09891462 | | | |  |  |  |  |  |  |  |  |  |  |
| 16 910.4044841 0.93253740 | | | |  |  |  |  |  |  |  |  |  |  |
| 17 905.2711099 0.56385643 | | | |  |  |  |  |  |  |  |  |  |  |
| 18 903.2705764 0.22098723 | | | |  |  |  |  |  |  |  |  |  |  |
| 19 841.2243723 6.86906068 | | | |  |  |  |  |  |  |  |  |  |  |
| 20 745.0967151 11.42711272 | | | |  |  |  |  |  |  |  |  |  |  |
| 21 609.6795609 18.17443984 | | | |  |  |  |  |  |  |  |  |  |  |
| 22 605.6684693 0.65790160 | | | |  |  |  |  |  |  |  |  |  |  |
| 23 604.7819779 0.14636578 | | | |  |  |  |  |  |  |  |  |  |  |
| 24 604.1862759 0.09849864 | | | |  |  |  |  |  |  |  |  |  |  |
| 25 603.7522395 0.07183818 | | | |  |  |  |  |  |  |  |  |  |  |
| 26 603.2166319 0.08871314 | | | |  |  |  |  |  |  |  |  |  |  |
| 27 602.6717138 0.09033538 | | | |  |  |  |  |  |  |  |  |  |  |
| 28 576.2005240 4.39230666 | | | |  |  |  |  |  |  |  |  |  |  |
| 29 557.3617461 3.26948295 | | | |  |  |  |  |  |  |  |  |  |  |
| 30 531.2003621 4.69378894 | | | |  |  |  |  |  |  |  |  |  |  |
| 31 509.4860821 4.08777582 | | | |  |  |  |  |  |  |  |  |  |  |
| 32 509.2891378 0.03865548 | | | |  |  |  |  |  |  |  |  |  |  |
| 33 505.1663711 0.80951398 | | | |  |  |  |  |  |  |  |  |  |  |
| 34 502.1604716 0.59503159 | | | |  |  |  |  |  |  |  |  |  |  |
| 35 500.1555056 0.39926800 | | | |  |  |  |  |  |  |  |  |  |  |
| 36 498.6872499 0.29355983 | | | |  |  |  |  |  |  |  |  |  |  |
| 37 496.1452505 0.50973819 | | | |  |  |  |  |  |  |  |  |  |  |
| 38 492.3128462 0.77243595 | | | |  |  |  |  |  |  |  |  |  |  |
| 39 489.0613894 0.66044526 | | | |  |  |  |  |  |  |  |  |  |  |
| 40 476.3162764 2.60603540 | | | |  |  |  |  |  |  |  |  |  |  |
| 41 470.0107532 1.32381016 | | | |  |  |  |  |  |  |  |  |  |  |
| 42 465.1920804 1.02522607 | | | |  |  |  |  |  |  |  |  |  |  |
| 43 460.7827686 0.94784757 | | | |  |  |  |  |  |  |  |  |  |  |
| 44 457.0682429 0.80613381 | | | |  |  |  |  |  |  |  |  |  |  |
| 45 451.7316193 1.16757697 | | | |  |  |  |  |  |  |  |  |  |  |
| 46 448.6348024 0.68554354 | | | |  |  |  |  |  |  |  |  |  |  |
| 47 447.4301420 0.26851693 | | | |  |  |  |  |  |  |  |  |  |  |
| 48 445.7519077 0.37508298 | | | |  |  |  |  |  |  |  |  |  |  |
| 49 443.9612510 0.40171599 | | | |  |  |  |  |  |  |  |  |  |  |
| 50 442.5056121 0.32787521 | | | |  |  |  |  |  |  |  |  |  |  |
| 51 441.5780127 0.20962433 | | | |  |  |  |  |  |  |  |  |  |  |
| 52 441.0252869 0.12517060 | | | |  |  |  |  |  |  |  |  |  |  |
| 53 440.1804832 0.19155448 | | | |  |  |  |  |  |  |  |  |  |  |
| 54 440.1547162 0.00585373 | | | |  |  |  |  |  |  |  |  |  |  |
| 55 440.1281973 0.00602490 | | | |  |  |  |  |  |  |  |  |  |  |
| 56 437.4481350 0.60892764 | | | |  |  |  |  |  |  |  |  |  |  |
| 57 435.2351398 0.50588745 | | | |  |  |  |  |  |  |  |  |  |  |
| 58 434.5770601 0.15120096 | | | |  |  |  |  |  |  |  |  |  |  |
| 59 434.2139573 0.08355315 | | | |  |  |  |  |  |  |  |  |  |  |
| 60 434.1470127 0.01541742 | | | |  |  |  |  |  |  |  |  |  |  |
| 61 434.1208045 0.00603671 | | | |  |  |  |  |  |  |  |  |  |  |
| 62 434.1175017 0.00076080 | | | |  |  |  |  |  |  |  |  |  |  |
| 63 434.1166245 0.00020207 | | | |  |  |  |  |  |  |  |  |  |  |
| 64 434.1164480 0.00004065 | | | |  |  |  |  |  |  |  |  |  |  |
| 65 434.1164305 0.00000404 | | | |  |  |  |  |  |  |  |  |  |  |
| 66 434.1164305 0.00000001 | | | |  |  |  |  |  |  |  |  |  |  |
| 67 434.1164305 0.00000000 | | | |  |  |  |  |  |  |  |  |  |  |
| Maximum Likelihood Estimates | | | | |  |  |  |  |  |  |  |  |  |
| Model: Censored Normal (cnorm) | | | | |  |  |  |  |  |  |  |  |  |
|  |  |  |  |  |  |  |  |  |  |  |  |  |  |
| Standard T for H0: | | | |  |  |  |  |  |  |  |  |  |  |
| Group Parameter Estimate Error Parameter=0 Prob > |T| | | | | | | |  |  |  |  |  |  |  |
|  |  |  |  |  |  |  |  |  |  |  |  |  |  |
| 1 Intercept 1.80502 0.03398 53.127 0.0000 | | | | | |  |  |  |  |  |  |  |  |
| Linear 0.00990 0.00064 15.549 0.0000 | | | | | |  |  |  |  |  |  |  |  |
|  |  |  |  |  |  |  |  |  |  |  |  |  |  |
| 2 Intercept 1.53927 0.03177 48.450 0.0000 | | | | | |  |  |  |  |  |  |  |  |
| Linear 0.00854 0.00057 14.995 0.0000 | | | | | |  |  |  |  |  |  |  |  |
|  |  |  |  |  |  |  |  |  |  |  |  |  |  |
| 3 Intercept 1.67562 0.12698 13.196 0.0000 | | | | | |  |  |  |  |  |  |  |  |
| Linear 0.02056 0.00248 8.307 0.0000 | | | | | |  |  |  |  |  |  |  |  |
|  |  |  |  |  |  |  |  |  |  |  |  |  |  |
| 1 Sigma 0.18614 0.00480 38.744 0.0000 | | | | | |  |  |  |  |  |  |  |  |
| 2 Sigma 0.18700 0.00403 46.451 0.0000 | | | | | |  |  |  |  |  |  |  |  |
| 3 Sigma 0.44941 0.01745 25.747 0.0000 | | | | | |  |  |  |  |  |  |  |  |
|  |  |  |  |  |  |  |  |  |  |  |  |  |  |
| Group membership | |  |  |  |  |  |  |  |  |  |  |  |  |
| 1 (%) 46.38836 2.23409 20.764 0.0000 | | | | | |  |  |  |  |  |  |  |  |
| 2 (%) 41.23408 2.28899 18.014 0.0000 | | | | | |  |  |  |  |  |  |  |  |
| 3 (%) 12.37755 1.27439 9.713 0.0000 | | | | | |  |  |  |  |  |  |  |  |
| **BIC= -480.04 (N=4226) BIC= -474.61 (N=1576) AIC= -445.12 ll= -434.12** | | | | | | | |  |  |  |  |  |  |
|  |  |  |  |  |  |  |  |  |  |  |  |  |  |
| Parameter estimates for adding risk factors | | | |  |  |  |  |  |  |  |  |  |  |
|  |  |  |  |  |  |  |  |  |  |  |  |  |  |
| 1.80502, 0.00990, 1.53927, 0.00854, 1.67562, 0.02056, | | | | | | |  |  |  |  |  |  |  |
| 0.18614, 0.18700, 0.44941, -0.11778, -1.32116 | | | | | |  |  |  |  |  |  |  |  |
|  |  |  |  |  |  |  |  |  |  |  |  |  |  |
|  |  |  |  |  |  |  |  |  |  |  |  |  |  |
| Parameter estimates | |  |  |  |  |  |  |  |  |  |  |  |  |
|  |  |  |  |  |  |  |  |  |  |  |  |  |  |
| 1.80502, 0.00990, 1.53927, 0.00854, 1.67562, 0.02056, | | | | | | |  |  |  |  |  |  |  |
| 0.18614, 0.18700, 0.44941, 46.38836, 41.23408, 12.37755 | | | | | | |  |  |  |  |  |  |  |
|  |  |  |  |  |  |  |  |  |  |  |  |  |  |
|  |  |  |  |  |  |  |  |  |  |  |  |  |  |
|  |  |  |  |  |  |  |  |  |  |  |  |  |  |
| Entropy = 0.735 | |  |  |  |  |  |  |  |  |  |  |  |  |

**REFERENCES**

1. Waldstein SR, Dore GA, Davatzikos C, Katzel LI, Gullapalli R, Seliger SL, et al. Differential Associations of Socioeconomic Status With Global Brain Volumes and White Matter Lesions in African American and White Adults: the HANDLS SCAN Study. Psychosom Med. 2017;79(3):327-35.

2. Beydoun MA, Hossain S, Chitrala KN, Tajuddin SM, Beydoun HA, Evans MK, et al. Association between epigenetic age acceleration and depressive symptoms in a prospective cohort study of urban-dwelling adults. J Affect Disord. 2019;257:64-73.

3. Beydoun MA, Shaked D, Tajuddin SM, Weiss J, Evans MK, Zonderman AB. Accelerated epigenetic age and cognitive decline among urban-dwelling adults. Neurology. 2020;94(6):e613-e25.

4. Tajuddin SM, Hernandez DG, Chen BH, Noren Hooten N, Mode NA, Nalls MA, et al. Novel age-associated DNA methylation changes and epigenetic age acceleration in middle-aged African Americans and whites. Clin Epigenetics. 2019;11(1):119.

5. Seeman T, Merkin SS, Crimmins E, Koretz B, Charette S, Karlamangla A. Education, income and ethnic differences in cumulative biological risk profiles in a national sample of US adults: NHANES III (1988-1994). Social science & medicine. 2008;66(1):72-87.

6. Alberti KG, Zimmet PZ. Definition, diagnosis and classification of diabetes mellitus and its complications. Part 1: diagnosis and classification of diabetes mellitus provisional report of a WHO consultation. Diabetic medicine : a journal of the British Diabetic Association. 1998;15(7):539-53.

7. Visser M, Kritchevsky SB, Newman AB, Goodpaster BH, Tylavsky FA, Nevitt MC, et al. Lower serum albumin concentration and change in muscle mass: the Health, Aging and Body Composition Study. Am J Clin Nutr. 2005;82(3):531-7.

8. Ridker PM. Cardiology Patient Page. C-reactive protein: a simple test to help predict risk of heart attack and stroke. Circulation. 2003;108(12):e81-5.

9. Expert Panel on Detection E, Treatment of High Blood Cholesterol in A. Executive Summary of The Third Report of The National Cholesterol Education Program (NCEP) Expert Panel on Detection, Evaluation, And Treatment of High Blood Cholesterol In Adults (Adult Treatment Panel III). Jama. 2001;285(19):2486-97.

10. Golden S, Boulware LE, Berkenblit G, Brancati F, Chander G, Marinopoulos S, et al. Use of glycated hemoglobin and microalbuminuria in the monitoring of diabetes mellitus. Evidence report/technology assessment. 2003(84):1-6.

11. Osei K, Rhinesmith S, Gaillard T, Schuster D. Is glycosylated hemoglobin A1c a surrogate for metabolic syndrome in nondiabetic, first-degree relatives of African-American patients with type 2 diabetes? The Journal of clinical endocrinology and metabolism. 2003;88(10):4596-601.

12. Seccareccia F, Pannozzo F, Dima F, Minoprio A, Menditto A, Lo Noce C, et al. Heart rate as a predictor of mortality: the MATISS project. American journal of public health. 2001;91(8):1258-63.

13. Lenfant C, Chobanian AV, Jones DW, Roccella EJ, Joint National Committee on the Prevention DE, Treatment of High Blood P. Seventh report of the Joint National Committee on the Prevention, Detection, Evaluation, and Treatment of High Blood Pressure (JNC 7): resetting the hypertension sails. Hypertension. 2003;41(6):1178-9.

14. Blackwell E, de Leon CF, Miller GE. Applying mixed regression models to the analysis of repeated-measures data in psychosomatic medicine. Psychosom Med. 2006;68(6):870-8.
